# Resistance Markers and Genetic Diversity in *Acinetobacter baumannii* Strains Recovered from Nosocomial Bloodstream Infections 

**DOI:** 10.3390/ijerph110201465

**Published:** 2014-01-28

**Authors:** Hanoch S. I. Martins, Maria Rosa Q. Bomfim, Rafaela O. França, Luiz M. Farias, Maria Auxiliadora R. Carvalho, José Carlos Serufo, Simone G. Santos

**Affiliations:** 1 Departamento de Microbiologia, Instituto de Ciências Biológicas, Universidade Federal de Minas Gerais, Antônio Carlos 6627, Pampulha 31207-901, Belo Horizonte, Minas Gerais, Brazil; E-Mails: hanoch.inacio@gmail.com (H.S.I.M.); mrqbomfim@gmail.com (M.R.Q.B.); faelafranca@yahoo.com.br (R.O.F.); macedo@icb.ufmg.br (L.M.F.); marc@icb.ufmg.br (M.A.R.C.); 2 Departamento de Parasitologia e Biologia, Universidade CEUMA, José Montello 1, 31067-120, São Luis do Maranhão, Brazil; E-Mail: mrqbomfim@gmail.com; 3 Departamento de Clínica Médica, Faculdade de Medicina, Universidade Federal de Minas Gerais, Professor Alfredo Balena 190, Santa Efigênia 30130-100, Belo Horizonte, MG, Brazil; E-Mail: serufo1@gmail.com

**Keywords:** *Acinetobacter baumannii*, bloodstream infections, genetic diversity, resistance markers

## Abstract

In this study, phenotypic and genotypic methods were used to detect metallo-β-lactamases, cephalosporinases and oxacillinases and to assess genetic diversity among 64 multiresistant *Acinetobacter baumannii* strains recovered from blood cultures in five different hospitals in Brazil from December 2008 to June 2009. High rates of resistance to imipenem (93.75%) and polymyxin B (39.06%) were observed using the disk diffusion (DD) method and by determining the minimum inhibitory concentration (MIC). Using the disk approximation method, thirty-nine strains (60.9%) were phenotypically positive for class D enzymes, and 51 strains (79.6%) were positive for cephalosporinase (AmpC). Using the E-test, 60 strains (93.75%) were positive for metallo-β-lactamases (MβLs). All strains were positive for at least one of the 10 studied genes; 59 (92.1%) contained *bla*_VIM-1_, 79.6% contained *bla*_AmpC_, 93.7% contained *bla*_OXA23_ and 84.3% contained *bla*_OXA51_. Enterobacteria Repetitive Intergenic Consensus (ERIC)-PCR analysis revealed a predominance of certain clones that differed from each other. However, the same band pattern was observed in samples from the different hospitals studied, demonstrating correlation between the genotypic and phenotypic results. Thus, ERIC-PCR is an appropriate method for rapidly clustering genetically related isolates. These results suggest that defined clonal clusters are circulating within the studied hospitals. These results also show that the prevalence of MDR *A. baumannii* may vary among clones disseminated in specific hospitals, and they emphasize the importance of adhering to appropriate infection control measures.

## 1. Introduction

Bacteremia caused by *Acinetobacter baumannii* is primarily an opportunistic nosocomial infection, most often acquired in intensive care units (ICUs). *A. baumannii* predominantly affects susceptible patients who have previously undergone invasive procedures [1]. These procedures include the placement of intravascular or urinary catheters, mechanical ventilation and previous surgery. Some cases are secondary to an undiagnosed infection caused by the use of an intravascular catheter or are due to translocation of intestinal bacteria [2,3].

*A. baumannii* has several mechanisms of drug resistance that have been reported globally as important causes of therapeutic failure in nosocomial infections. This resistance of samples to different classes of antimicrobials compromises the treatment of infected patients. In contrast to community-acquired strains, isolates from hospital environments exhibit high rates of antimicrobial resistance and are often resistant to multiple drugs.

The general mechanisms of antibiotic resistance in *Acinetobacter* spp. include enzyme-mediated resistance, genetic adaption, efflux pumps, porin mutations, changes in the structure of outer membrane components and production of acquired carbapenem-hydrolyzing class D β-lactamases (CHDLs) [4,5,6]. *Acinetobacter* spp. have acquired a variety of β-lactamases, the production of which affects porins in the outer membrane, making *A. baumannii* impermeable and, thus, resistant to antibiotics [7].

Imipenem and meropenem were traditionally the most effective antimicrobials against the *A. baumannii* complex [8], but the carbapenem-resistant *A. baumannii* complex (CRAB) has become common worldwide [9]. Since the emergence of carbapenem as the therapeutic of choice for treating severe infections caused by *Acinetobacter* spp., the use of this class of drugs has been threatened by both the increasing incidence of β-lactamases that can hydrolyze these antibiotics and the spread of multidrug-resistant (MDR) clones.

The aim of this study was to evaluate antimicrobial resistance; the production of metallo-β-lactamase, oxacillinase and cephalosporinase enzymes; and the genetic diversity in *A. baumannii* strains isolated from patients at different hospitals in Belo Horizonte in the Brazilian state of Minas Gerais.

## 2. Experimental Section

### 2.1. Bacterial Strains, Identification and Susceptibility Testing

This study evaluated 64 strains of MDR *A. baumannii* isolated from blood cultures between December 2008 and June 2009. All samples were previously identified using the GN Card of the bioMerieuxVITEK2 ^®^ System. The strains were obtained from hospitals that provide general assistance, emergency services and outpatient care.

For *in vitro* antimicrobial susceptibility testing, we used the disk diffusion (DD) and minimum inhibitory concentration (MIC) agar dilution techniques to test the following antimicrobials and doses: 30 μg ceftazidime (CAZ), 30 μg cefepime (CPM), 30 μg amikacin (AMI), 10 μg gentamycin (GEN), 5 μg ciprofloxacin (CIP), 5 μg levofloxacin (LVX), 30 μg aztreonam (ATM), 10 μg imipenem (IP), 10 μg meropenem (MER) and 300 μg polymyxin B (POL). All disks were obtained from Laborclin (Sao Paulo, Brazil). Results were interpreted according to the critical points recommended by the Clinical and Laboratory Standards Institute (CLSI) in 2011 (M100-S21) [4]. The reference strains *A. baumannii* ATCC 19606, *P. aeruginosa* ATCC 27853 and *E. coli* ATCC 25922 were used as controls for all phenotypic tests.

### 2.2. Phenotypic Detection of Class B (Metallo-β-Lactamase) Enzymes Related to Resistance

To study metallo-β-lactamases (MβLs), two different phenotyping methods were used: Disk Approximation (DA) and MβL E-test ^®^.

#### 2.2.1. Disk Approximation (DA) Test

The DA test was performed and interpreted as previously described, with some modifications [10,11,12]. The MβL inhibitors used in this study were 2-mercaptopropionic acid (MPA), 2-mercaptoacetic acid (MAA) and ethylenediaminetetraacetic acid (EDTA) (Sigma, St. Louis, MO, USA) at concentrations of 2.11 mM, 4.14 mM and 100 mM, respectively. The substrates used in this study were disks containing 30 μg CAZ and 10 μg imipenem (IP) (Laborclin ^®^, Pinhais, Brazil). Strains that exhibited antimicrobial synergism between CAZ and/or IP and at least one of the employed inhibitors were considered MβL-producing strains. MβL-producing samples displayed MβL distortion and a broadened zone of inhibition of bacterial growth in the region of diffusion of the MβL inhibitor. In negative tests, the zone of inhibition of bacterial growth was unchanged.

#### 2.2.2. MΒLE-Test ^®^

For the MβL E-test ***^®^***, strips containing a gradient of imipenem ranging from 4–256 μg/mL at one end, and a gradient ranging from 1–64 μg/mL of imipenem associated with EDTA (IPI) at the other end, were added to plates inoculated with the bacterial suspension and then incubated at 35 °C for 24 h. Samples was considered MβL-producing when the ratio of the MIC (μg/mL) for IP to the MIC for IPI was greater than or equal to 8 and when there was a decrease of three dilutions between IP and IPI. The onset of deformation of the ellipse (*i.e.*, the appearance of a ghost area) was also considered indicative of the production of MBL and ESBL.

### 2.3. Polymerase Chain Reaction for the Detection of β-Lactamase Genes

Bacterial DNA was extracted using the thermal lysis method followed by centrifugation for 30 s at 9,000 rpm and 4 °C [13,14]. The DNA in the supernatant was quantified using a Nanodrop and stored in a freezer until use. Genotypic detection was performed for the following genes: *bla*_SPM-1_, *bla*_IMP-1_, *bla*_GIM-1_, *bla*_SIM-1_, *bla*_VIM-1_, *bla*_AmpC_, *bla*_OXA23_, *bla*_OXA24_, *bla*_OXA51_ and *bla*_OXA58_ (Table 1). PCR was performed in a final volume of 25 µL with 12.5 µL Master Mix (Promega ^®^, Madison, WI, USA), 1.5 µL primers, 100 ng bacterial DNA and nuclease-free water to reach a volume of 25 µL. PCR was performed according to the conditions described by the author for each set of primers used.

**Table 1:** Primers used for detection of β-lactamase genes in *A. baumannii* strains.

Genes	Primers	Product (pb)	Reference
*bla*_SPM-1_	5’-AAAATCTGGGTACGCAAACG-3’ 5’-ACATTATCCGCTGGAACAGG-3’	271 pb	[15]
*bla*_IMP-1_	5’-GAATAGAGTGGCTTAACTCTC-3’ 5’-AGATAACCTAGTGGTTTGG-3’	189 pb	[15]
*bla*_VIM-1_	5’-CGAATGCGCAGCACCAG-3’ 5’-CTGGTGCTGCGCATTCG-3’	390 pb	[15]
*bla*_GIM-1_	5’-TCGACACACCTTGGTCTGAA-3’ 5’-AACTTCCAACTTTGCCATGC-3’	477 pb	[15]
*bla*_SIM-1_	5’-TACAAGGGATTCGGCATCG-3’ 5’-TAATGGCCTGTTCCCATGTG-3’	571 pb	[16]
*bla*_AmpC_	5’-CAGTAGCGAGACTGCGCA-3’ 5’-ATAACCACCCAGTCACGC-3’	631 pb	[17,18]
*bla*_OXA23_	5’-GATCGGATTGGAGAACCAGA-3’ 5’-ATTTCTGACCGCATTTCCAT-3’	501 pb	[6]
*bla*_OXA24_	5’-GGTTAGTTGGCCCCCTTAAA-3’ 5’-AGTTGAGCGAAAAGGGGATT-3’	246pb	[6]
*bla*_OXA51_	5’-TAATGCTTTGATCGGCCTTG-3’ 5’-ATTTCTGACCGCATTTCCAT-3’	353 pb	[6]
*bla*_OXA58_	5’-AAGTATTGGGGCTTGTGCTG-3’ 5’-CCCCTCTGCGCTCTACATAC-3’	599 pb	[6]

#### PCR Product Analysis

PCR products were analyzed using 2% agarose gel electrophoresis in TBE buffer (2 mM EDTA, 10 mM Trisborate, pH 8.0) at 100 volts for 2 h. The gels were stained with ethidium bromide (0.5 mg/mL).

### 2.4. Characterization of the Genetic Profiles of the Strains

Characterization of the genetic profiles of the bacterial strains was performed using DNA polymorphism analysis after amplification using PCR with primers for conserved sequences from the Enterobacteria Repetitive Intergenic Consensus region (ERIC-PCR). *A. baumannii* strains that were positive for any MβL genes were analyzed using ERIC-PCR with the ERIC1 (5’ TGTAAGCTCCTGGGGATTCAC 3’) and ERIC2 (5’ AAGTAAGTGACTGGGGTGAGCG 3’) primers, as previously described [19]. Electrophoresis of the PCR products was performed on a 1.5% agarose gel at 90 volts for approximately 2 h. A dendrogram was used to determine the genetic relationships between the strains using NTSYS version 2.1 (Exeter Software, New York, NY, USA) using the DICE similarity coefficient and the unweighted pair group method with arithmetic mean (UPGMA). In all analyses, we used the strain *A. baumannii* ATCC 19606 as a negative control and previously sequenced *A. baumannii* strains encoding the analyzed genes as positive controls.

### 2.5. Ethical Considerations

This study was approved by the Research Ethics Committees of the participating hospitals and the COEP/UFMG (ETIC 614/08).

## 3. Results and Discussion

### 3.1. Antimicrobial Susceptibility Profiles

**Figure 1:**
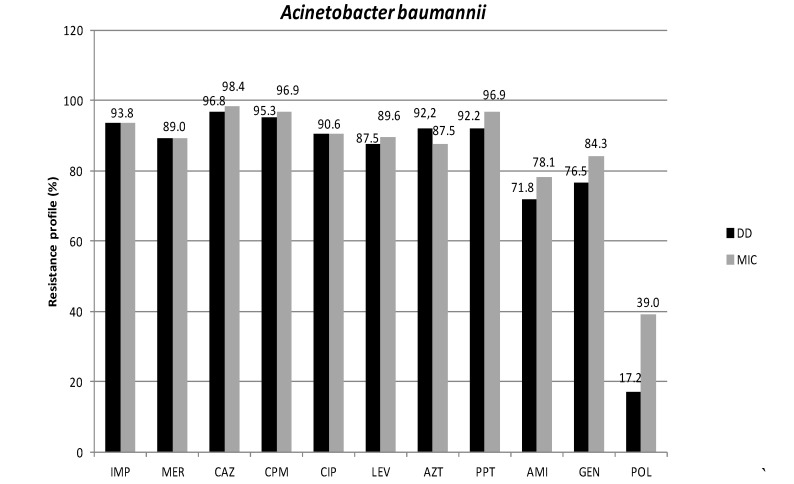
Resistance profiles of 64 *A. baumannii* strains isolated from blood cultures from patients at five hospitals in this study. IP: Imipenem; MER: Meropenem; CAZ: Ceftazidime; CPM: Cefepime; ATM: Aztreonam; PTZ: Piperacillin/Tazobactam; CIP: Ciprofloxacin; LEV: Levofloxacin; AMI: Amikacin; GEN: Gentamycin; POL: Polymyxin B; DD: disk diffusion; MIC: minimum inhibitory concentration.

In this study, antimicrobial susceptibility testing of *A. baumannii* strains using disk diffusion (DD) and minimum inhibitory concentration (MIC) by agar dilution showed high rates of resistance to all tested drugs (Figure 1). The results from the two methods used (DD and MIC) were similar for almost all tested drugs, but were notably different for polymyxin B (POL). The lowest resistance to POL was obtained with DD, a result that may be due to poor diffusion of this antimicrobial agent in agar [4]. Comparison of the discordant results from POL by the two methods confirms the lowest accuracy of DD for testing this drug [4].

In Brazil, as in many parts of the world, outbreaks of infections caused by MDR *Acinetobacter* have been reported frequently. The rates of resistance to carbapenems have been considerably higher in Brazil than in other countries; some studies found rates of up to 100.0% resistance in the evaluated isolates [20,21,22].

The resistance to carbapenems was found to be high in all hospitals evaluated in our study, with the resistance profile ranging from 71.4% to 100%. Notably, resistance to carbapenems (both imipenem and meropenem) was recorded as 100% in two hospitals. For amikacin and gentamycin, the aminoglycosides studied here, the resistance percentages varied between hospitals, from 57.1% to 87.5%. Resistance to 4th generation cephalosporins, represented by cefepime, was extremely high, with 0% sensitivity in four of the five hospitals studied. The resistance profile to quinolones (ciprofloxacin and levofloxacin) ranged from 100% resistance in one hospital to 62.5% in another.

A study that performed molecular characterization of MDR strains of *Acinetobacter* spp. in Porto Alegre, Brazil, reported that 69% of strains were resistant to carbapenems [19]. MβL-producing strains of *A. baumannii* are often resistant to all treatment options. Thus, other drugs, such as polymyxin B, are frequently used therapeutically, despite their toxicity [22]. In the current study, 39% of these strains were resistant to polymyxin B, with MIC_50_ and MIC_90_ values of 2 µg/mL and 8 µg/mL, respectively.

Of the 64 samples of *A. baumannii* identified by automated methods, 20 (31.25%) strains showed full and intermediate resistance profile to all tested drugs, including polymyxin, and 23 (35.9%) were sensitive to only this antimicrobial agent in the evaluated hospitals.

### 3.2. Phenotypic Detection of Enzymes Related to Resistance

In our study, high numbers of MβL-producing *A. baumannii* were detected with the combinations IMI + EDTA and IP + MPA, with positive results in 14% (9/64) and 25% (16/64) of strains, respectively. According to the E-test, 60 strains (63.75%) were positive for MβL production.

The high antimicrobial resistance to carbapenems that is observed in *A. baumannii* strains is caused by multiple mechanisms. These mechanisms include decreased permeability of the outer membrane; losses or structural alterations of porins; activity of efflux pumps that reduce the concentration of the antimicrobial agent inside the bacteria; and/or the action of β-lactam hydrolyzing enzymes, including carbapenemases such as cephalosporins, penicillins and carbapenems [9]. Several acquired β-lactamases that belong to Ambler Class B, also known as MβLs, or Class D, also known as oxacillinases, have been identified in these organisms [23,24,25].

The results of this study revealed differences between the MβL tests used. This variation may be due to methodological aspects of the tests, the bacterial species under study or the types of metallo-enzymes involved.

Studies comparing phenotypic tests for the detection of MβLs have demonstrated that these tests are not suitable for *Acinetobacter* isolates [26]. There is considerable disagreement in the literature regarding the best technique for phenotypic detection of these enzymes. Some studies report that 2-MPA provides higher sensitivity, although interpretation is difficult and subjective, while other studies suggest that EDTA can be used successfully. The action of EDTA on the permeability of the cell wall can accelerate the breakdown of IMI and can decrease the expression of membrane proteins [27].

### 3.3. Detection of β-Lactamase Genes

**Figure 2:**
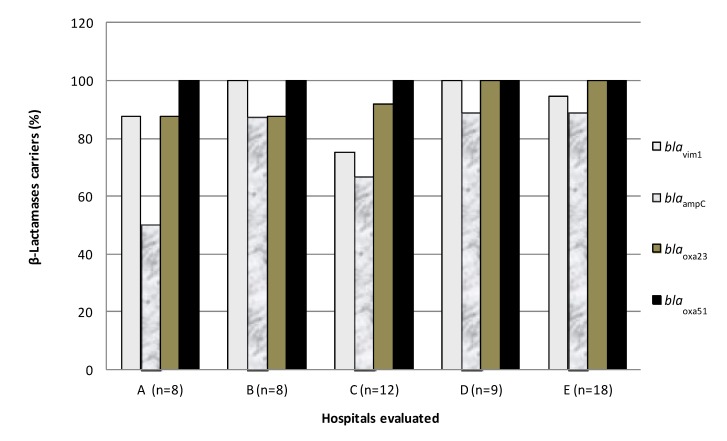
Percentages of *bla*_OXA51 _positive *A. baumannii* strains, carrying genes encoding other oxacillinases, MβLs, and cephalosporinases by hospital.

All strains of *A. baumannii* (64/64), previously identified by automated phenotypic methods, were positive for at least one of the genes studied. Fifty-nine samples (92.1%) contained a PCR product for a gene fragment of *bla*_VIM-1_, 51 (79.6%) contained the gene *bla*_AmpC_, 60 (93.7%) contained the *bla*_OXA23_ gene, and 55 (85.9%) *bla*_OXA51_, considered a species-specific gene. The genes *bla*_IMP-1_, *bla*_SIM-1_, *bla*_GIM-1_, *bla*_SPM-1_, *bla*_OXA24_ and *bla*_OXA58_ were not identified in any of the *A. baumannii* strains evaluated in this study. Only samples containing the gene *bla*_OXA51_ (n = 55) were considered for further molecular analysis. The distributions of MβL-, oxacillinase- and cephalosporinase- encoding genes by evaluated hospital are shown in Figure 2.

The results of the phenotypic and genotypic analyses applied to *A. baumannii* showed 37.5% agreement with respect to the production of MβLs when using the DA test, and 63.75% agreement when using the E-test. Table 2 shows the frequencies of different combinations of β-lactamase genes in *A. baumannii* strains (n = 55), with *bla*_VIM-1,_ *bla*_AmpC, _*bla*_OXA23 _and *bla*_OXA51 _as the most frequent combination.

Lineages of OXA-23-positive *A. baumannii* have spread around the world to places including France, Bulgaria, Iran, the United Arab Emirates, Tunisia, Brazil, Australia and other countries. In Brazil, *A. baumannii* producing OXA-23 was first detected in 2003 in Curitiba, a city in the South, and quickly spread to neighboring states. Since that time, an increasing incidence of infections caused by carbapenem-resistant strains of this bacterium has been observed [20,21,22].

A study conducted in Sao Paulo city evaluating bloodstream infection by *A. baumannii* in a university hospital, showed that of the 29 samples resistant to carbapenems detected in patients in the ICU, 21 (72.4%) were positive for the OXA-23 enzyme [26,27].

*Acinetobacter* spp. has a variety of known β-lactamases, but oximino-β-lactamase resistance, often attributable to the enzyme AmpC, is increasingly observed in this opportunistic pathogen. The enzyme is normally expressed at low levels and is not inducible, but over expression occurs with the joint insertion upstream of an insertion element (ISA*ba1*) in *A. baumannii*, which provides an effective promoter for *bla*_AmpC_. Plasmids carrying genes for AmpC β-lactamases frequently carry multiple other resistances, including genes for resistance to drugs such as aminoglycosides, chloramphenicol, quinolones, tetracyclines, sulfonamides and trimethoprim [28,29].

**Table 2:** Frequencies of different combinations of β-lactamases genes in *A. baumannii* strains (n = 55).

Genes	Frequency	%
*bla*_VIM-1-_ *bla*_AmpC-_ *bla*_OXA23-_ *bla*_OXA51_	37	67.3
*bla*_VIM-1-_ *bla*_OXA23-_ *bla*_OXA51_	11	20.0
*bla*_AmpC-_ *bla*_OXA23-_ *bla*_OXA51_	3	5.5
*bla*_VIM-1-_ *bla*_AmpC-_ *bla*_OXA51_	2	3.6
*bla*_OXA23-_ *bla*_OXA51_	1	1.8
*bla*_VIM-1- _ *bla*_Oxa51_	1	1.8

### 3.4. Genotypic Characterization of A. Baumannii Strains

For the genotypic analysis of DNA using ERIC primers, all *A. baumannii* strains that were positive for *bla*_OXA51 _genes (n = 55) were included. Genotypic analysis using ERIC-PCR produced an average of three to 12 fragments per *A. baumannii* strain (determined by visual inspection, data not shown). 

**Figure 3:**
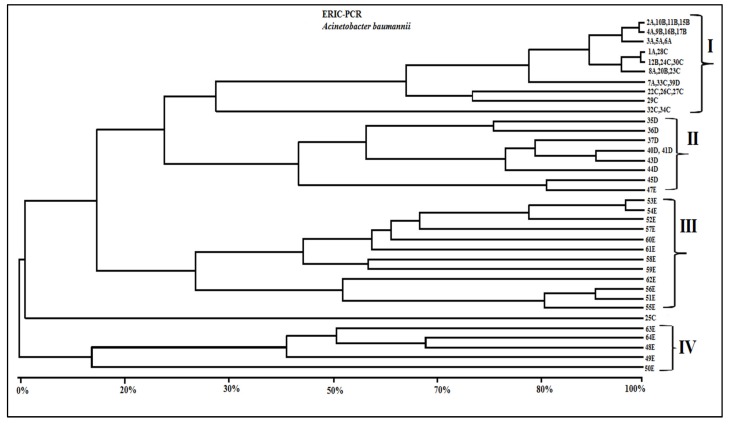
Dendrogram of genetic similarities showing the relationships between the analyzed *A. baumannii* strains (n = 55). The dendrogram was derived from DICE similarity coefficients, implemented using the NTSYS program. The construction was made using clustering with the UPGMA method.

In the genetic relationship study, only distinct, reproducible, well-resolved ERIC fragments in the size range of 70–1,500 bp were scored as present (1) or absent (0), and from band scores a binary data matrix was constructed in the NTSYS-pc 2.02 software package [30]. The matrix of DICE similarity coefficients generated a phenetic dendrogram derived from the unweighted pair group method with arithmetic mean (UPGMA) algorithm. A visual inspection of this dendrogram showed heterogeneous profiles, in which the degree of similarity varied from 15%–100%. Figure 3 shows the grouping of the results according to phenotypic similarity among profiles produced by ERIC-PCR. Notably, some samples from Hospitals A, B and C showed similar band patterns and were grouped together. An exception to this is sample 25C, which showed a different profile than all other isolates. In group II, were clustered samples from Hospital D, with the exception of sample 39D which was grouped with isolates of group I. Groups III and IV were formed by isolates from Hospital E, with heterogeneous clonal profiles for all 18 isolates. Isolate 47E was grouped with the samples of group II. The results obtained from each hospital, including the drug resistance profiles, the β-Lactamases genotypes and phenotypes detected, and clone cluster assignments obtained with dendrogram, are shown in Table 3.

ERIC-PCR profiles identified the following strains circulating in Hospital A, profile I: 1A, 2A, 3A, 4A, 5A, 6A, 7A and 8A; and in Hospital B, profile I included strains 9B, 10B, 11B, 12B, 15B, 16B, 17B and 20B. Moreover, in Hospital C, profile I included strains 22C, 23C, 24C, 26C, 27C, 28C, 29C, 30C, 32C, 33C and 34C. The isolate 25C, which showed a different profile by ERIC-PCR, was not grouped with other strains; this sample was sensitive only to polymyxin B. This result indicates that a different clone of resistance is likely circulating in Hospital C, though further research is needed to confirm this hypothesis. 

Regarding Hospital D, almost all isolates were included in group II, integrating the following strains: 35D, 36D, 37D, 40D, 41D, 43D, 44D and 45D. Isolate 39D was integrated into group I and expressed the *bla*_VIM-1-_*bla*_OXA23_-*bla*_OXA51_ and *bla*_AmpC_ genes, demonstrating resistance to all antibiotics evaluated in this study.

Isolates from Hospital E were distributed among groups II, III and IV. Strains 51E, 52E, 53E, 54E, 55E, 56E, 57E, 58E, 59E, 60E and 61E were integrated into profile III, while group IV included the strains 48E, 49E, 50E, 63E and 64E. Only the isolate 47E was included in group II. A predominance of circulating clones differing from each other but expressing the genes *bla*_VIM-1_-*bla*_OXA23_-*bla*_OXA51_ and *bla*_AmpC _was observed in Hospital E (Table 3). This result is interesting because this hospital, which is in the Hospital Foundation of Minas Gerais network, receives patients from almost all municipalities in the state of Minas Gerais. However, all hospitals evaluated showed strains that demonstrated resistance profiles for all genes surveyed. This result suggests that the same clone is circulating among these hospitals. The data also indicate that these strains may be disseminated by different routes, such as health professionals and the transfer of patients between hospitals.

**Table 3:** Profiles obtained by ERIC-PCR, β-Lactamase results and antimicrobial resistance phenotypes in evaluated *A. baumannii* strains.

Isolate/ Hospital	ERIC-PCR Groups	Resistance Genes	Etest MBL	Phenotypic Profile Resistance
1A	I	*bla*_AmpC, _ *bla*_Oxa23, _ *bla*_Oxa51_	+	IM, ME, CA, CP, CI, LE, AZ, PT, AM, GE
2A	I	*bla*_VIM-1, _ *bla*_AmpC, _ *bla*_Oxa23, _ *bla*_Oxa51_	+	IM, ME, CA, CP, CI, LE, AZ, PT, AM, GE, PO
3A	I	*bla*_VIM-1, _ *bla*_Oxa23, _ *bla*_Oxa51_	+	IM, ME, CA, CP, AZ, PT, AM, GE, PO
4A	I	*bla*_VIM-1, _ *bla*_AmpC, _ *bla*_Oxa23, _ *bla*_Oxa51_	+	IM, ME, CA, CP, CI, LE, PT
5A	I	*bla*_VIM-1, _ *bla*_Oxa23, _ *bla*_Oxa51_	+	IM, ME, CA, CP, CI, LE, AZ, PT, AM, GE, PO
6A	I	*bla*_VIM-1, _ *bla*_Oxa23, _ *bla*_Oxa51_	+	IM, ME, CA, CP, AZ, PT, AM, GE, PO
7A	I	*bla*_VIM-1, _ *bla*_Oxa23, _ *bla*_Oxa51_	+	IM, ME, CA, CP, CI, LE, AZ, PT, AM, GE, PO
8A	I	*bla*_VIM-1, _ *bla*_AmpC_ *, bla*_Oxa51_	+	IM, ME, CA, CP, CI, LE, AZ, PT, AM, GE, PO
9B	I	*bla*_VIM-1, _ *bla*_AmpC, _ *bla*_Oxa23, _ *bla*_Oxa51_	+	IM, ME, CA, CP, CI, LE, AZ, PT, AM, GE
10B	I	*bla*_VIM-1, _ *bla*_AmpC, _ *bla*_Oxa23, _ *bla*_Oxa51_	+	IM, ME, CA, CP, CI, LE, AZ, PT, AM, GE, PO
11B	I	*bla*_VIM-1, _ *bla*_AmpC, _ *bla*_Oxa23, _ *bla*_Oxa51_	+	IM, ME, CA, CP, CI, LE, AZ, PT, AM, GE, PO
12B	I	*bla*_VIM-1, _ *bla*_Oxa23, _ *bla*_Oxa51_	+	IM, ME, CA, CP, CI, LE, AZ, PT, AM, GE
15B	I	*bla*_VIM-1, _ *bla*_AmpC, _ *bla*_Oxa23, _ *bla*_Oxa51_	+	IM, ME, CA, CP, CI, LE, AZ, PT, AM, GE, PO
16B	I	*bla*_VIM-1, _ *bla*_AmpC, _ *bla*_Oxa23, _ *bla*_Oxa51_	+	IM, ME, CA, CP, CI, LE, AZ, PT, AM, GE, PO
17B	I	*bla*_VIM-1, _ *bla*_AmpC, _ *bla*_Oxa23, _ *bla*_Oxa51_	+	IM, ME, CA, CP, CI, LE, AZ, PT, AM, GE, PO
20B	I	*bla*_VIM-1, _ *bla*_AmpC, _ *bla*_Oxa51_	+	IM, ME, CA, CP, CI, LE, AZ, PT, AM, GE
22C	I	*bla*_VIM-1, _ *bla*_AmpC, _ *bla*_Oxa23, _ *bla*_Oxa51_	+	IM, ME, CA, CP, CI, LE, AZ, PT, AM, GE
23C	I	*bla*_VIM-1, _ *bla*_AmpC, _ *bla*_Oxa51_	+	IM, ME, CA, CP, CI, LE, AZ, PT, AM, GE, PO
24C	I	*bla*_VIM-1, _ *bla*_Oxa51_	+	IM, ME, CA, CP, CI, LE, AZ, PT, AM, GE
25C	Ungrouped	*bla*_Oxa23, _ *bla*_Oxa51_	+	IM, ME, CA, CP, CI, LE, AZ, PT, AM, GE
26C	I	*bla*_VIM-1, _ *bla*_AmpC, _ *bla*_Oxa23, _ *bla*_Oxa51_	+	IM, ME, CA, CP, AZ, PT, AM, GE, PO
27C	I	*bla*_VIM-1, _ *bla*_AmpC, _ *bla*_Oxa23, _ *bla*_Oxa51_	+	IM, ME, CA, CP, LE, AZ, PT, AM
28C	I	*bla*_AmpC, _ *bla*_Oxa23, _ *bla*_Oxa51_	−	IM, ME, CA, CP, CI, LE, AZ, PT, AM, GE, PO
29C	I	*bla*_VIM-1, _ *bla*_AmpC, _ *bla*_Oxa23, _ *bla*_Oxa51_	+	IM, ME, CA, CP, CI, LE, AZ, PT, AM, GE, PO
30C	I	*bla*_VIM-1, _ *bla*_Oxa23, _ *bla*_Oxa51_	+	IM, ME, CA, CP, CI, LE, AZ, PT, AM, GE, PO
32C	I	*bla*_VIM-1, _ *bla*_AmpC, _ *bla*_Oxa23, _ *bla*_Oxa51_	+	IM, ME, CA, CP, CI, LE, AZ, PT, AM, GE, PO
33C	I	*bla*_VIM-1, _ *bla*_Oxa23, _ *bla*_Oxa51_	+	IM, ME, CA, CP, CI, LE, AZ, PT, AM, GE
34C	I	*bla*_VIM-1,_ *bla*_AmpC, _ *bla*_Oxa23, _ *bla*_Oxa51_	+	IM, ME, CA, CP, CI, LE, AZ, PT, AM, GE
35D	II	*bla*_VIM-1, _ *bla*_AmpC, _ *bla*_Oxa23, _ *bla*_Oxa51_	+	IM, ME, CA, CP, CI, LE, AZ, PT, AM, GE, PO
36D	II	*bla*_VIM-1, _ *bla*_AmpC, _ *bla*_Oxa23, _ *bla*_Oxa51_	+	IM, ME, CA, CP, CI, LE, AZ, PT, AM, GE
37D	II	*bla*_VIM-1, _ *bla*_AmpC, _ *bla*_Oxa23, _ *bla*_Oxa51_	+	IM, ME, CA, CP, CI, LE, AZ, PT, AM, GE, PO
39D	I	*bla*_VIM-1, _ *bla*_Oxa23, _ *bla*_Oxa51_	+	IM, ME, CA, CP, CI, LE, AZ, PT, AM, GE, PO
40D	II	*bla*_VIM-1, _ *bla*_AmpC, _ *bla*_Oxa23, _ *bla*_Oxa51_	+	IM, ME, CA, CP, CI, LE, AZ, PT, AM, GE, PO
41D	II	*bla*_VIM-1, _ *bla*_AmpC, _ *bla*_Oxa23, _ *bla*_Oxa51_	+	IM, ME, CA, CP, CI, LE, AZ, PT, AM, GE
43D	II	*bla*_VIM-1, _ *bla*_AmpC, _ *bla*_Oxa23, _ *bla*_Oxa51_	+	IM, ME, CA, CP, CI, LE, AZ, PT, AM, GE, PO
44D	II	*bla*_VIM-1, _ *bla*_AmpC, _ *bla*_Oxa23, _ *bla*_Oxa51_	+	IM, ME, CA, CP, CI, LE, AZ, PT, AM, GE
45D	II	*bla*_VIM-1, _ *bla*_AmpC, _ *bla*_Oxa23, _ *bla*_Oxa51_	+	IM, ME, CA, CP, CI, AZ, PT, AM, GE
47E	II	*bla*_VIM-1, _ *bla*_AmpC, _ *bla*_Oxa23, _ *bla*_Oxa51_	+	IM, ME, CA, CP, CI, LE, AZ, PT, AM, GE
48E	IV	*bla*_VIM-1, _ *bla*_AmpC, _ *bla*_Oxa23, _ *bla*_Oxa51_	+	IM, ME, CA, CP, CI, LE, AZ, PT, AM, GE
49E	IV	*bla*_VIM-1, _ *bla*_AmpC, _ *bla*_Oxa23, _ *bla*_Oxa51_	+	IM, ME, CA, CP, CI, LE, AZ, PT, AM, GE, PO
50E	IV	*bla*_VIM-1, _ *bla*_AmpC, _ *bla*_Oxa23, _ *bla*_Oxa51_	+	IM, ME, CA, CP, CI, LE, AZ, PT, AM, GE
50E	IV	*bla*_VIM-1, _ *bla*_AmpC, _ *bla*_Oxa23, _ *bla*_Oxa51_	+	IM, ME, CA, CP, CI, LE, AZ, PT, AM, GE
51E	III	*bla*_VIM-1, _ *bla*_Oxa23, _ *bla*_Oxa51_	+	IM, ME, CA, CP, CI, LE, AZ, PT, AM, GE
52E	III	*bla*_VIM-1, _ *bla*_AmpC, _ *bla*_Oxa23, _ *bla*_Oxa51_	+	IM, ME, CA, CP, CI, LE, AZ, PT, AM, GE
53E	III	*bla*_VIM-1, _ *bla*_AmpC, _ *bla*_Oxa23, _ *bla*_Oxa51_	+	IM, ME, CA, CP, CI, LE, AZ, PT, AM, GE
54E	III	*bla*_VIM-1, _ *bla*_AmpC, _ *bla*_Oxa23, _ *bla*_Oxa51_	+	IM, ME, CA, CP, CI, LE, AZ, PT, AM, GE
55E	III	*bla*_Ampc, _ *bla*_Oxa23, _ *bla*_Oxa51_	+	IM, ME, CA, CP, CI, LE, AZ, PT, AM, GE
56E	III	*bla*_VIM-1, _ *bla*_Oxa23, _ *bla*_Oxa51_	+	IM, ME, CA, CP, CI, LE, AZ, PT, AM, GE
57E	III	*bla*_VIM-1, _ *bla*_AmpC, _ *bla*_Oxa23, _ *bla*_Oxa51_	+	IM, CA, CP, CI, LE, AZ, PT
58E	III	*bla*_VIM-1, _ *bla*_AmpC, _ *bla*_Oxa23, _ *bla*_Oxa51_	+	IM, ME, CA, CP, CI, LE, AZ, PT, AM
59E	III	*bla*_VIM-1, _ *bla*_AmpC, _ *bla*_Oxa23, _ *bla*_Oxa51_	+	IM, ME, CA, CP, CI, LE, AZ, PT, AM,
60E	III	*bla*_VIM-1, _ *bla*_AmpC, _ *bla*_Oxa23, _ *bla*_Oxa51_	+	IM, ME, CA, CP, CI, LE, AZ, PT, AM, GE
61E	III	*bla*_VIM-1, _ *bla*_AmpC, _ *bla*_Oxa23, _ *bla*_Oxa51_	+	IM, ME, CA, CP, CI, LE, AZ, PT, AM, GE
62E	III	*bla*_VIM-1, _ *bla*_AmpC, _ *bla*_Oxa23, _ *bla*_Oxa51_	+	IM, ME, CA, CP, CI, LE, AZ, PT
63E	IV	*bla*_VIM-1, _ *bla*_AmpC, _ *bla*_Oxa23, _ *bla*_Oxa51_	+	IM, ME, CA, CP, CI, LE, AZ, PT
64E	IV	*bla*_VIM-1, _ *bla*_AmpC, _ *bla*_Oxa23, _ *bla*_Oxa51_	−	IM, CA, CP, CI, LE, AZ, PT, AM, GE

Notes: IM: imipenem; ME: meropenem; CA: ceftazidime; CP: cefepime; CI: ciprofloxacin; LE: levofloxacin; AZ: aztreonam; PT: piperacicllin/tazobactam; AM: amikacin; GE: gentamicin; PO: polymyxin B.

## 4. Conclusions

The results of this study show that the prevalence of MDR *A. baumannii* may vary among clones disseminated in specific hospitals, and they emphasize the importance of adherence to appropriate infection control measures. In addition, early detection of strains producing enzymes such as MβLs and oxacillinases is extremely important. The detection of MDR strains in clinical laboratories may help reduce the dissemination of such enzyme-producing strains because most of the genes encoding these enzymes are localized in highly mobile genetic elements. The possible existence of a predominant genotype among the analyzed strains reinforces the importance of intervention measures, such as contact precautions, to reduce the chances of cross-infection, to better match the physical area of hospitals and clinics and to improve adherence to basic measures of nosocomial infection control.
